# Editorial: Fat metabolism and deposition in poultry: physiology, genetics, nutrition Volume II

**DOI:** 10.3389/fphys.2023.1264512

**Published:** 2023-08-01

**Authors:** Sami Dridi

**Affiliations:** Center of Excellence for Poultry Science, University of Arkansas, Fayetteville, AR, United States

**Keywords:** fat metabolism, thermo-conditioning, omics, chickens, ducks

As we described in our previous editorial for volume I of this Research Topic, modern broilers have been subjected to extensive genetic selection to improve feed efficiency and achieve high growth rate and muscle yield. However, this has concomitantly led to excessive fat deposition, which has increased the incidence of metabolic and cardiac disorders along with elevated sudden death rate (Squires and Summers, 1993; Chen et al., 2017; Olkowski et al., 2020).

In continuity to the volume I, where we gathered several elegant papers describing the ontology of lipid metabolism in fat tissue and omics-associated factors regulating hepatic fat metabolism in chickens, we invited outstanding authors and pioneer scientists to further provide and highlight additional knowledges through innovative scientific research.

Andrieux and others have optimized the embryonic thermo-conditioning that increased foie gras production without quality alteration and this effect was mediated via hepatic metabolism reprogramming (Andrieux et al.).

Luo and co-workers determined, by using gas chromatography-mass spectrometry (GC-MS) and untargeted metabolomics, the main volatile organic compounds (VOC) in chicken breast muscle and abdominal fat (Luo et al.). They identified nine VOCs in both tissues and found that amino acids are the main precursors of 1-octen-3-ol, (E,E)-2, 4-nonadienal, and heptanal in chicken meat, while fatty acids are the main precursors of diethyl disulfide. Hexanal, however, can be synthesized from amino acids and small amounts of fatty acids as precursors.

Hicks and colleagues investigated the hepatic cell cycle transcriptional profile and network during the metabolic switch in broilers. They used high throughput RNA-seq to identify the transcriptome profile and differences in and between the liver of E18 embryos and 2-day post hatch chicks fed or not fed from hatch. Hundred of differentially expressed genes were identified between the abovementioned groups and I encourage the readers to check this manuscript (Hicks et al.).

Kong et al., on the other hand, used integrated high throughput metabolomics and lipidomics to evaluate the alterations of flavor precursors in chicken breast muscle-affected with white striping. The white striping myopathy, first appeared in 2009 (Bauermeister et al., 2009), is visually characterized by white fat striations parallel to muscle fibers on the breast (Kuttappan et al., 2013), and is of increasing concern to the industry and consumer because of the unappealing appearance and altered flavor of the affected filets. Although its etiology is still unknown, white striping has been reported to have histological lesions, including myo-degeneration and necrosis, lymphocyte, fat, and macrophage infiltration, fibrosis, and lipidosis (Trocino et al., 2015). Here, Kong and co-workers found that white striping-affected breast was distinguished from normal unaffected breast by a decreased level of E-nose, and four volatile compounds (o-xylene, benzene, 1,3-dimethyl, 2-heptanone and 6-methyl and acetic acid and ethyl ester) (Kong et al., 2022). Lipidomics analyses revealed increased levels of neutral lipid and decreased phospholipids in white striping-affected muscle. Metabolomics analyses showed an alteration of 16 metabolites, including water-soluble flavor precursors such as AMP, GDP-fucose, and L-arginine in white striping-affected muscles.

In summary, the studies reported in the current Research Topic used managerial manipulation (thermo-conditioning) and cutting-edge techniques and provided some mechanistic understanding of hepatic lipid metabolism and white striping myopathy. However, although the Research Topic is a research hot spot, four published papers is quite low and represent a limitation, and I hope that in the future we will attract more investigators and more manuscripts.
